# Supplementary material for: Meta-Analysis and Gene Set Enrichment Relative to ER Status Reveal Elevated Activity of MYC and E2F in the “Basal” Breast Cancer Subgroup
Source: PLoS One. 2009 Mar 9;4(3):e4710. doi: 10.1371/journal.pone.0004710 (PMC2650420; doi:10.1371/journal.pone.0004710)
Supplement: Dataset S1 — GSEA results from first screen. The file contains a zipped archive of the HTML results of the second GSEA screen. Reports for enriched or depleted datasets may be accessed from the “index.html” link within the my_analysis.GseaPreranked.1219975950625.rpt.zip ZIP archive. Enrichment plots are available for each of the 50 most enriched and depleted gene sets. (13.32 MB ZIP) [file pone.0004710.s001.zip › my_analysis.GseaPreranked.1219975950625.rpt.zip!//CHR12Q12.html]

Details for gene set CHR12Q12[GSEA]

|  || Dataset | zSco.ERneg.to.ERpos\_collapsed |
| Phenotype | NoPhenotypeAvailable |
| Upregulated in class | na\_neg |
| GeneSet | CHR12Q12 |
| Enrichment Score (ES) | -0.4217796 |
| Normalized Enrichment Score (NES) | -1.583394 |
| Nominal p-value | 0.01510574 |
| FDR q-value | 0.13280523 |
| FWER p-Value | 0.999 |
Table: GSEA Results Summary

  

Fig 1: Enrichment plot: CHR12Q12      
 Profile of the Running ES Score & Positions of GeneSet Members on the Rank Ordered List

  

| PROBE | GENE SYMBOL | GENE\_TITLE | RANK IN GENE LIST | RANK METRIC SCORE | RUNNING ES | CORE ENRICHMENT || 1 | KRT6B |  |  | 34 | 6.501 | 0.0767 | No |
| 2 | SHMT2 |  |  | 308 | 4.920 | 0.1161 | No |
| 3 | KRT7 |  |  | 1262 | 3.180 | 0.0831 | No |
| 4 | TUBA6 |  |  | 1802 | 2.689 | 0.0753 | No |
| 5 | KRT83 |  |  | 2433 | 2.233 | 0.0550 | No |
| 6 | KRT85 |  |  | 2480 | 2.211 | 0.0785 | No |
| 7 | KRT3 |  |  | 4129 | 1.460 | -0.0278 | No |
| 8 | PRPH |  |  | 4649 | 1.269 | -0.0514 | No |
| 9 | ACCN2 |  |  | 4973 | 1.165 | -0.0615 | No |
| 10 | ADCY6 |  |  | 5032 | 1.142 | -0.0520 | No |
| 11 | COL2A1 |  |  | 5293 | 1.050 | -0.0587 | No |
| 12 | ADAMTS20 |  |  | 5758 | 0.892 | -0.0828 | No |
| 13 | TIMELESS |  |  | 5760 | 0.892 | -0.0720 | No |
| 14 | AQP2 |  |  | 6294 | 0.717 | -0.1034 | No |
| 15 | HOXC11 |  |  | 7294 | 0.385 | -0.1739 | No |
| 16 | VDR |  |  | 7985 | 0.110 | -0.2245 | No |
| 17 | MLL2 |  |  | 8604 | -0.133 | -0.2695 | No |
| 18 | TUBA3 |  |  | 8675 | -0.160 | -0.2728 | No |
| 19 | STAT2 |  |  | 8780 | -0.200 | -0.2782 | No |
| 20 | HOXC8 |  |  | 8981 | -0.305 | -0.2895 | No |
| 21 | GPD1 |  |  | 9243 | -0.427 | -0.3040 | No |
| 22 | YAF2 |  |  | 9328 | -0.472 | -0.3045 | No |
| 23 | IRAK4 |  |  | 9645 | -0.642 | -0.3205 | No |
| 24 | HOXC5 |  |  | 9738 | -0.690 | -0.3190 | No |
| 25 | PTK9 |  |  | 10177 | -0.943 | -0.3405 | No |
| 26 | HOXC13 |  |  | 10703 | -1.318 | -0.3640 | No |
| 27 | PUS7L |  |  | 10900 | -1.473 | -0.3608 | No |
| 28 | HOXC10 |  |  | 11345 | -1.878 | -0.3713 | No |
| 29 | PDZRN4 |  |  | 12016 | -2.580 | -0.3903 | Yes |
| 30 | DAZAP2 |  |  | 12274 | -2.932 | -0.3740 | Yes |
| 31 | ORMDL2 |  |  | 12304 | -2.969 | -0.3400 | Yes |
| 32 | TEGT |  |  | 12420 | -3.138 | -0.3104 | Yes |
| 33 | RND1 |  |  | 12421 | -3.139 | -0.2721 | Yes |
| 34 | HOXC4 |  |  | 12463 | -3.204 | -0.2361 | Yes |
| 35 | PFDN5 |  |  | 12640 | -3.509 | -0.2066 | Yes |
| 36 | SLC38A1 |  |  | 12718 | -3.652 | -0.1679 | Yes |
| 37 | HOXC6 |  |  | 12743 | -3.723 | -0.1244 | Yes |
| 38 | CD63 |  |  | 12872 | -4.071 | -0.0844 | Yes |
| 39 | TARBP2 |  |  | 13087 | -4.776 | -0.0423 | Yes |
| 40 | PRKAG1 |  |  | 13118 | -4.904 | 0.0152 | Yes |
Table: GSEA details [plain text format]

  

Fig 2: CHR12Q12: Random ES distribution      
 Gene set null distribution of ES for **CHR12Q12**
